# Dysregulated neural coding in the vagus nerve during long sepsis

**DOI:** 10.1016/j.bbih.2025.101043

**Published:** 2025-06-19

**Authors:** Joshua J. Strohl, Tomás S. Huerta, Sergio Robbiati, Patricio T. Huerta

**Affiliations:** a Institute of Bioelectronic Medicine, Feinstein Institutes for Medical Research, Northwell Health, Manhasset, NY, USA; b Department of Molecular Medicine, Zucker School of Medicine at Hofstra/Northwell, Hempstead, NY, USA; c Elmezzi Graduate School of Molecular Medicine, Northwell Health, Manhasset, NY, USA

**Keywords:** Sepsis, Vagus nerve, Cytokine, Inflammation, Inflammatory reflex, Machine learning, Mouse, Neurophysiology, Cuff electrode

## Abstract

Sepsis is a life-threatening condition characterized by organ dysfunction resulting from the body's unbalanced and excessive response to an infection. ‘Long sepsis’ (LS) is an emerging concept referring to persistent sequelae experienced by long-term sepsis survivors, which include cognitive impairment, immune dysfunction, high cardiovascular risk, fatigue, and depression. Here, we investigated the role of the vagus nerve, a key component of the inflammatory reflex, in a mouse model of LS. Six weeks after cecal ligation and puncture (CLP) or sham surgery, we performed electrophysiological recordings from the cervical vagus nerve in anesthetized male mice. We found that LS mice exhibited significantly higher baseline vagal activity compared to controls, with elevated firing rates during both respiratory bursts and inter-burst intervals. Control mice showed clear increases in vagal activity following systemic administration of pro-inflammatory cytokines, tumor necrosis factor (TNF) and interleukin-1β (IL-1β), but LS mice displayed markedly dysregulated responses. LS mice showed altered firing dynamics, with many vagal units decreasing rather than increasing their activity after cytokine stimulation. Using a naïve Bayes decoder, we demonstrated that LS disrupted the neural code in the vagus nerve, significantly altering its activity pattern in response to cytokine signals. These results suggest that LS fundamentally alters vagus nerve function, with elevated baseline activity and diminished responsiveness to inflammatory signals. This neurophysiological dysregulation may contribute to the persistent multi-organ dysfunctions observed in long sepsis survivors, suggesting a potential role for vagal signaling in sepsis outcomes.

## Highlights

• Neurophysiological recordings show increased vagus nerve activity in long sepsis.
• The vagal response to inflammatory cytokine challenge is blunted.
• Machine learning reveals disrupted cytokine encoding by the vagus nerve.

## 1 Introduction

Sepsis is a leading global health challenge, known for its insidious onset and devastating impact. It is a clinical syndrome of organ dysfunction triggered by a dysregulated host response to infection (Singer et al., 2016). Sepsis not only stands as one of the foremost causes of mortality but also leaves survivors facing chronic health issues; a condition we term ‘long sepsis’ (LS) (Strohl et al., 2024). The immunology of sepsis has been intensely studied (for review, see van der Poll et al., 2021), revealing a dynamic process initiated by infectious species that release pathogen-associated molecular patterns. These molecules activate innate immune cells (macrophages, neutrophils), which then secrete massive levels of inflammatory cytokines such as tumor necrosis factor (TNF), interleukin-1β (IL-1β), and interleukin-6 (IL-6) into circulation. This cytokine surge contributes to acute septic symptoms such as fever, increased heart rate, and low blood pressure. Counter-intuitively, during this hyperinflammatory event, a period of severe immune suppression begins, which peaks shortly after the hyperinflammatory episode. This immunosuppression is characterized by T-cell exhaustion and anergy, immune cell apoptosis, and increased secretion of anti-inflammatory cytokines such as interleukin-10 (IL-10) and transforming growth factor-β (TGF-β) (Torres et al., 2022). Immunosuppression increases the susceptibility to secondary infections and hinders pathogen clearance, despite the possibly concurrent high levels of inflammation. The balance between pro- and anti-inflammatory responses, including their timing and intensity, varies significantly between individuals, contributing to sepsis heterogeneity. At the resolution of these severely dysregulated immune responses, some patients may recover to a normal health status but many sepsis survivors experience long-term sequalae. This population will suffer cognitive impairment and brain dysfunction, immune anergy (which increases the susceptibility to infection), increased risk of cardiovascular events, and dysfunctions in other organs, leading to an overall increase in the likelihood of hospital readmission and death for months to years after discharge (Angus et al., 2001; Annane and Sharshar, 2015; Iwashyna et al., 2010; Iwashyna et al., 2012; Manabe and Heneka, 2022; Semmler et al., 2013; van der Poll et al., 2021). This constellation of syndromes emerges as a distinct condition that we term long sepsis (LS). Importantly, the persistent organ disruptions linked to LS have increased steadily as advances in critical care have led to decreased in-hospital mortality and more sepsis survivors (Iwashyna et al., 2012; Mostel et al., 2020). Our own work supports these clinical observations and demonstrates lasting cognitive impairment and brain network dysfunction in mouse models of LS (Chavan et al., 2012; Strohl et al., 2024).

Amid this critical backdrop, the vagus nerve emerges as a key player in modulating the body's response to sepsis. Though often referred to as a singular entity, the vagus nerve actually arises as a dual structure (tenth cranial nerve) from the left and right sides of the brainstem. It then ramifies into a vast array of fibers that innervate the heart, gastrointestinal tract, liver, lungs, bronchi, trachea, larynx, pharynx, and the external ear (Berthoud and Neuhuber, 2000; Ma et al., 2025; Pavlov and Tracey, 2012; Yuan and Silberstein, 2016). Sensory vagal neurons continually survey the physiological state of internal organs and deliver their signals to the central nervous system (CNS) via the nucleus tractus solitarius (Cunningham et al., 2008; van der Kooy et al., 1984; Zittel et al., 1999). This creates a visceral sensory function comparable to the classical senses of vision, sound, touch, smell and taste (Jin et al., 2024; Prescott and Liberles, 2022; Schappe et al., 2024; Shaffer et al., 2023; Tamari et al., 2024; Zhao et al., 2022). The sensory neurons express molecular receptors that detect several chemical stimuli, such as cholecystokinin, glucagon-like-peptide-1 and ghrelin, as well as mechanical stimuli like stretch or tension (Browning et al., 2017; Waise et al., 2018). Moreover, the vagus nerve contains motor (efferent) neurons that integrate sensory vagal signals with numerous CNS inputs and transmit efferent signals to the periphery, thus closing the sensory-motor loop for the visceral sense (Tracey, 2002).

The vagus nerve is a key component of the inflammatory reflex, a neural pathway that modulates immune response to injury and pathogen invasion. The sensory arc of the inflammatory reflex is activated by cytokines that are released by immune cells, whereas the motor arc suppresses the production of cytokines in target organs, such as the spleen (Borovikova et al., 2000; Pavlov and Tracey, 2012; Tracey, 2002). The motor arc has been well-delineated: vagal fibers terminate in the celiac ganglion, activating neurons of the splenic nerve, which in turn stimulate a specialized subset of T cells that release acetylcholine (Chavan et al., 2017; Rosas-Ballina et al., 2011). Splenic macrophages express α-7 nicotinic acetylcholine receptors, the activation of which inhibits inflammatory cytokine secretion (Wang et al., 2003). The sensory arc has been elucidated through electrophysiological recordings of vagal afferents, starting with seminal studies showing that IL-1β could activate vagal fibers (Niijima, 1996; Niijima et al., 1995; Watkins et al., 1995). Cervical vagus recordings of adult mice have demonstrated cytokine-specific neurograms in sensory vagal fibers following administration of the proinflammatory cytokines TNF and IL-1β (Silverman et al., 2018; Steinberg et al., 2016; Zanos et al., 2018). This inflammatory reflex may play a crucial role in the progression and resolution of sepsis, particularly in the development of LS. Indeed, preclinical findings suggest that the vagus nerve contributes to immunosuppression in sepsis as vagotomy has been found to protect the inflammatory response to lipopolysaccharide challenge (Rana et al., 2018). While the inflammatory reflex is crucially involved in the interactions between the nervous and immune systems, there are other neuroimmune circuits. For instance, stimulation of the afferent fibers of the vagus nerve can induce anti-inflammatory effects independently of acetylcholine-releasing T cells, which has been suggested to have an efferent pathway through the splanchnic nerves (Komegae et al., 2018; McAllen et al., 2022; Murray et al., 2021).

In this study, we examine the neurophysiological activity of the vagus nerve in a mouse model of LS. Our focus is on the left vagus nerve, as it contains predominantly afferent fibers and fewer cardiac efferent fibers compared to the right side. This anatomical distinction is the basis for the therapeutic use of left vagus nerve stimulation (Binnie, 2000). Additionally, we have deliberately chosen to study the long-term consequences of sepsis, by focusing on a timepoint when acute physiological disturbances and elevations in pro-inflammatory markers have resolved (Valdés-Ferrer et al., 2013). To this end, mice were subjected to the cecal ligation and puncture (CLP) procedure and allowed to recover; only survivors were examined six weeks following the initial insult. This model reliably induces polymicrobial sepsis, recapitulating both the hyper-inflammatory and hypo-inflammatory phases that are characteristic of clinical sepsis. Unlike the endotoxemia model, which causes a transient elevation in certain pro-inflammatory cytokines, CLP leads to a prolonged period of cytokine release and weight loss, both of which resolve by our recording timepoint (Dejager et al., 2011; Stevens et al., 2017; Valdés-Ferrer et al., 2013). We believe this approach is well-suited to studying the chronic effects of LS on the vagus nerve.

Our electrophysiological recordings reveal that vagal signaling in LS mice is highly dysregulated. Specifically, we observe constitutively increased vagal activity and an altered response to injected cytokines. This altered response diminishes our ability to decipher responses to systemically injected cytokines, indicating a fundamental disruption of the inflammatory reflex. These findings may shed light on the persistent immune and neural dysfunctions seen in sepsis survivors and could guide the development of novel therapeutic approaches targeting vagal signaling in LS.

## 2 Materials & methods

### *2.1* Ethics statement

Animal experiments followed National Institutes of Health (NIH) Guidelines under protocols approved by the Feinstein Institutes for Medical Research Institutional Animal Care and Use Committee (IACUC). Our Animal Research Program is registered with the Department of Health and Human Services (DHHS), Office of Laboratory Animal Welfare (OLAW), U.S. Department of Agriculture (USDA #21R0107), Public Health Service (PHS #A3168–01) and New York State Department of Health (NYSDOH #A- 060). All procedures followed ARRIVE guidelines and were approved by the Feinstein's IACUC (protocol #2023–003).

### *2.2* Animals

C57BL/6 J mice (*n* = 75, male, 6 weeks-of-age) were purchased from the Jackson Laboratory (Bar Harbor, ME) and maintained on a 12-h reverse circadian cycle (dark 9:00–21:00, light 21:00–9:00) with ad libitum access to food and water. Mice acclimated for one week before experiments began and were initially housed 5 per cage. All experiments occurred during the dark phase.

### *2.3* Surgical groups

Mice underwent either CLP (*n* = 50, LS group) or sham surgery (*n* = 25, control group, CON) at 8 weeks of age. Mice were anesthetized with 2.5 % isoflurane (maintained at 2.0 %) and placed on a heating pad. After shaving and sterilizing the abdomen (with isopropyl alcohol and betadine solution), an incision was made to expose the cecum. For CLP, the cecum's end was ligated using 4–0 non-absorbable suture (Ethicon, Somerville, NJ), punctured with a 22G needle, and ∼1 mm of stool extruded. The perforated cecum was returned to the abdominal cavity, and the muscle wall was closed with absorbable sutures and the skin with wound clips. For sham surgery, the cecum was exposed, gently manipulated, and returned without puncture. At the conclusion of the surgery, the mouse was placed into a new home cage which was kept over a heating pad until the mouse was fully mobile again. All animals received 0.5 mL saline resuscitation, 0.03 mg/kg Buprenex (Reckitt Benckiser Healthcare, Slough, UK), and 0.5 mg Primaxin antibiotic in 0.5 mL saline (Merck, Kenilworth, NJ). Mice were monitored daily for 2 weeks post-surgery and, while all CON mice made a full recovery within a week, about half of LS mice (*n* = 23) perished and the rest (n = 27) recovered fully, which is the expected survival rate for this procedure.

### 2.4 Isolation of the vagus nerve

Mice were fasted 4 h prior to recordings. Isolation and recordings of the vagus nerve were performed under isoflurane anesthesia. Mice were induced at 2.5 % isoflurane, laid in the supine position, and maintained at ∼2.0 % isoflurane while the vagus nerve was being isolated and placed onto the recording electrode. At this point, mice were maintained at 1.25 % isoflurane for the duration of the recording. A midline incision was made in the neck and the salivary glands were separated with blunt forceps. The left cervical vagus nerve was identified by the pulsing of the carotid artery, and the nerve was gently isolated from the carotid bundle using fine forceps. The sheath of connective tissue surrounding the nerve was removed using fine forceps, and the nerve was placed over the recording electrode. A platinum ground wire was placed underneath the left salivary gland.

### 2.5 Vagus nerve recordings

Neurophysiological recordings were obtained using a 3-lead AirRay Research Cuff electrode (CorTec GmbH, Freiburg, DE) for compound action potentials (CAPs) and a silver wire electrode placed under the chest cavity skin for electrocardiogram signals. Both electrodes and the ground wire were connected to an Omnetics EIB-18 and Digital Lynx 4SX digital data acquisition system (Neuralynx, Bozeman, MT). Signals were digitized at a sampling rate of 32 kHz and recorded using Cheetah software (Neuralynx, Bozeman, MT).

Each recording session lasted 1 h, consisting of 20 min baseline (BL) followed by two intraperitoneal cytokine injections at 20 min and 40 min. These recording durations and analysis windows were based on established protocols showing robust vagal responses within 20 min of cytokine administration (Steinberg et al., 2016; Zanos et al., 2018). The experiment was counterbalanced for cytokine order: half the mice received TNF (50 μg per mouse) followed by IL-1β (350 ng per kg), while the other half received IL-1β followed by TNF. These doses were selected based on previous work from our group (Steinberg et al., 2016). Mice were euthanized after recordings. Recordings were excluded from analysis if damage to the vagus nerve was observed during the procedure or if no spikes were detected during the recording session.

### 2.6 Data analysis

Recordings were analyzed in MATLAB with the algorithm described by Zanos et al. (2018), online at https://public.feinsteininstitute.org/cbem/PNAS%20Submission/. A band pass filter (160–3000 Hz) was applied followed by an adaptive threshold to isolate spikes and identify respiratory bursts. Spikes were sorted using t-distributed stochastic neighbor embedding (t-SNE) with the Toolbox for Dimensionality Reduction (https://lvdmaaten.github.io/drtoolbox/) in MATLAB. Unsupervised classification used density-based spatial clustering of applications with noise (DBSCAN). All clusters were manually inspected and those corresponding to cardiac signals removed. Isolated unit firing rates were exported to Origin software (OriginLab, Northampton, MA) for graph generation and statistical comparisons.

### 2.7 Analysis of waveforms and firing rates

Waveforms were examined with custom written MATLAB scripts. For each unit, the average waveform was calculated to obtain peak-to-peak amplitude and half-height time width. To quantify the firing rates, we only used the second 10-min BL period to reduce interference from nerve manipulation. For the analysis of cytokine responses, we used a strict inclusion criterion by excluding fibers with BL firing rates higher than one standard deviation of the population average or with zero firing for more than 5 consecutive minutes post-injection. For analysis of cytokine ratios (presented in Fig. 5D), we computed the summed frequency of all CAPs from each mouse for the last 10 min of BL (termed *f*_BL_), TNF (termed *f*_TNF_), and IL-1β (termed *f*_IL1_), and computed the ratios for TNF (defined as [*f*_TNF_ – *f*_BL_]/[*f*_TNF_ + *f*_BL_]) and IL-1β (defined as [*f*_IL1_– *f*_BL_]/[*f*_IL1_ + *f*_BL_]).

### 2.8 Bayesian decoding

Bayesian decoding was performed in MATLAB using the technique described in Zanos et al. (2018). The data used to train the algorithm for each mouse and data to be decoded consisted of the firing rate of each individual unit at each second of the recording, smoothed with a 30-point Gaussian-weighted moving average filter in MATLAB. For each recording, the decoder was trained using a subset of the data acquired and one of three classification categories: BL, post-TNF injection, and post-IL-1β injection. The remaining data in the recording was then sent into the trained algorithm to be decoded, and probabilities were given for at each timepoint for the three possible classes.

### *2.9* Statistics

Statistical testing was done with Origin software. Normally distributed data (confirmed by Shapiro-Wilk normality test) were analyzed with the two-tailed Student's *t*-test (termed *t*-test, henceforth). Non-parametric distributions used the Mann-Whitney *U* test (termed MW test, henceforth) or the Kolmogorov-Smirnov test (termed KS test, henceforth). Statistical significance was set at *P* < 0.05.

## 3 Results

### 3.1 Vagus nerve recordings and unit isolation

**Fig. 1:**
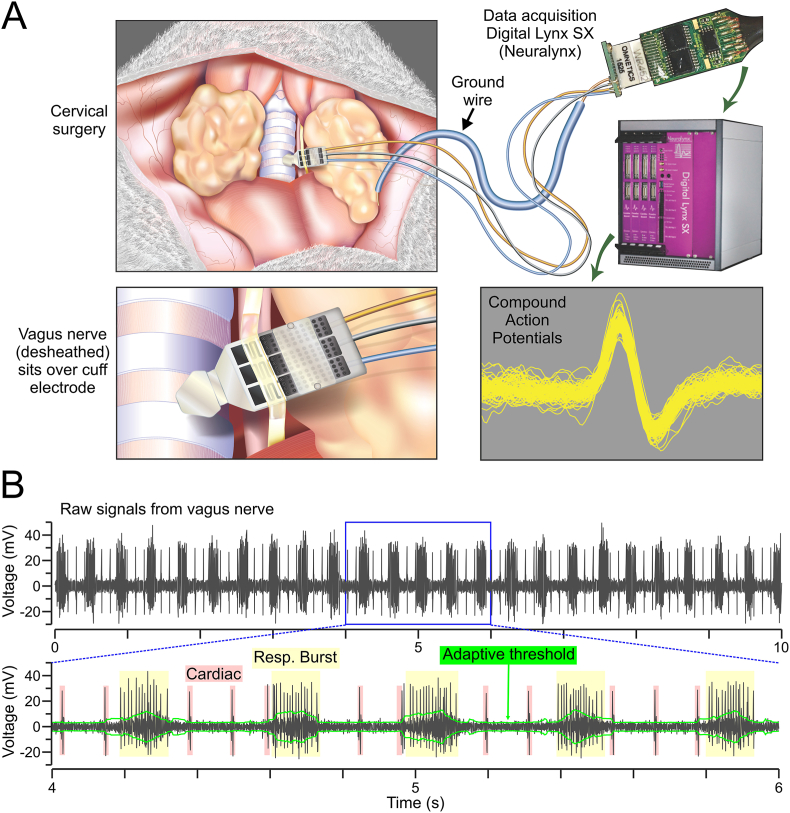
Neurophysiological recordings in the cervical vagus nerve. **(A)** The cervical surgery involves a neck incision, isolation of the vagus nerve and removal of the surrounding sheath (*top left*). The desheathed nerve is placed over the recording electrode (*bottom left*) and the ground wire is placed under the left salivary gland. The electrode's leads and ground wire are connected to an electronic interface board (EIB, Omnetics) that carries the signal to the data acquisition system (*top right*), which records compound action potentials (*bottom right*). **(B)** Raw signals showing a 10-s interval (*top*) and an expanded 2-s view (*bottom*), which contains both neural and non-neural components. An adaptive threshold (in green) is applied for isolating the CAPs, accounting for the effect of respiration that leads to respiratory bursts (“Resp. Burst” in yellow). These are recurring periods with higher background noise and robust vagus nerve's activity. Cardiac signals (in red) are also recorded. (For interpretation of the references to color in this figure legend, the reader is referred to the Web version of this article.)

We studied how long sepsis (LS) affects vagus nerve activity and its immune-encoding properties. Mice underwent either CLP (LS group) or sham surgery (control group, CON) followed by a 6-week recovery period. Before recording, we found no differences between groups in body weight (mean ± SEM, CON = 31.9 ± 0.9 g, LS = 30.3 ± 0.6 g, *P* = 0.13, *t*-test) or body temperature (mean ± SEM, CON = 36.9 ± 0.1 °C, LS = 37.0 ± 0.3 °C, *P* = 0.42, MW test). Following established protocols for detecting cytokine-related signals in the vagus nerve (Silverman et al., 2018; Steinberg et al., 2016; Zanos et al., 2018), we recorded from the left cervical vagus nerve with a CorTec cuff electrode to capture CAPs, which represent groups of fibers in the vagus nerve firing action potentials in unison (Fig. 1A) (Silverman et al., 2018; Steinberg et al., 2016). The recordings contained neural activity, respiratory events, and cardiac signals. During ‘respiratory bursts’, the firing rate of CAPs increased along with the background noise. To account for the increased noise, we used an adaptive threshold which increased its value during respiratory bursts, allowing us to accurately capture the neural activity while minimizing recording artifacts originating from non-neural sources (Fig. 1B).

**Fig. 2:**
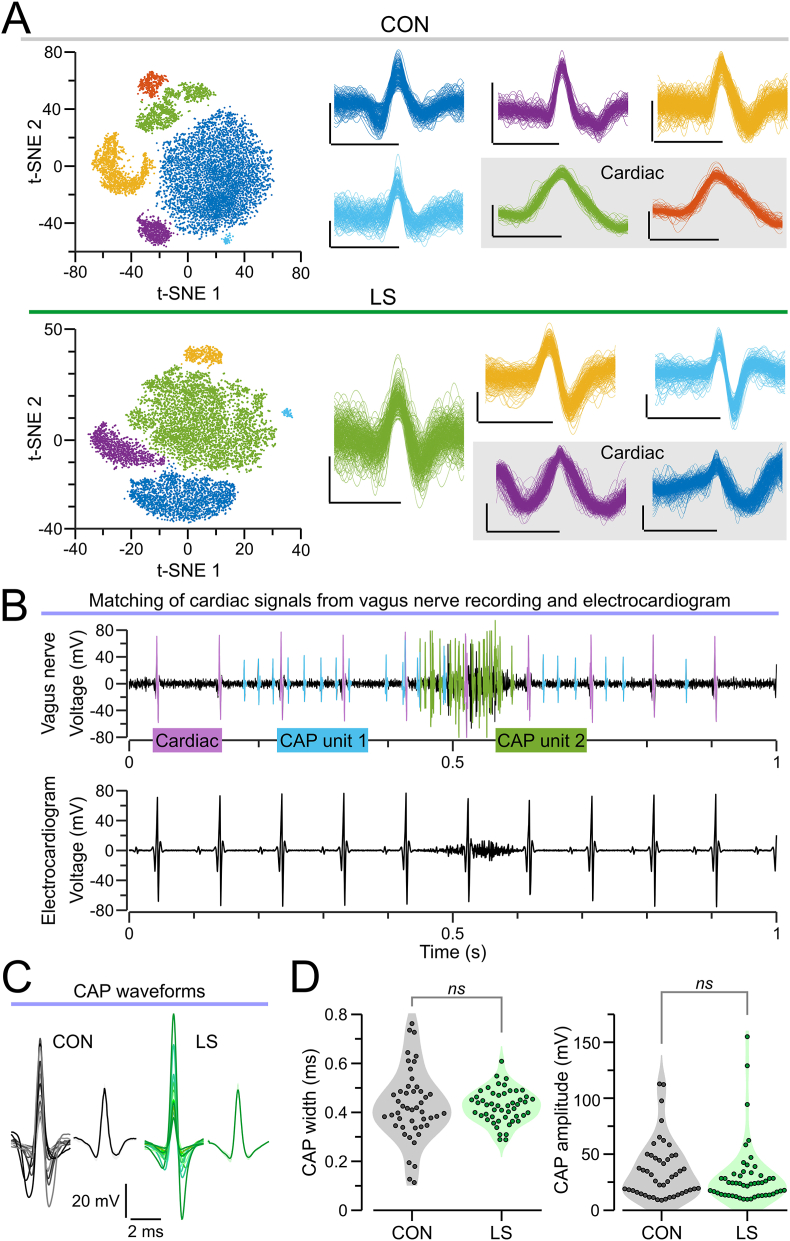
Clustering and waveform analysis of compound action potentials. **(A)***Top*, representative t-SNE plot with CAP clusters (*left*) and waveforms of sorted units (*right*) for the vagus nerve of a single CON mouse. *Bottom*, same as above for a single LS mouse. Each color corresponds to an isolated vagal unit seen as a distinct cluster in the t-SNE plot (*at left*) and as overlaid waveforms (*at right*). Clusters corresponding to cardiac signals (waveforms shown in grey boxes) are discarded from further analysis. **(B)** Representative 1-s recording showing simultaneous traces from the vagus nerve cuff electrode (*top*) and the recording from the electrocardiogram electrode (*bottom*). The vagus nerve recording contains two distinct CAPs (blue and green), and a cardiac component (violet) that was excluded from all further analysis. The electrocardiogram trace shows cardiac signals that temporally align with the cardiac component in the vagus recording but contains no CAPs, confirming the neural origin of the CAP signals. **(C)** CAP waveforms (*n* = 15) from a single CON unit and a single LS unit, with their average waveform (mean ± SEM) shown to the right of each set. **(C)** Violin plots for the width of the CAP waveforms (*left*, *P* = 0.879, *t*-test) and their amplitude (*right*, *P* = 0.204, KS test). Each dot represents a sorted CAP unit; *ns*, non-significant. (For interpretation of the references to color in this figure legend, the reader is referred to the Web version of this article.)

When recording CAPs from the vagus nerve, multiple groups of fibers can be recorded from each animal, each generating a characteristic waveform. We sorted these waveforms into distinct units using t-SNE followed by DBSCAN clustering (Fig. 2A) and removed any clusters corresponding to cardiac signals (Fig. 2A and B). Each of the remaining clusters represented a distinct CAP unit. Notably, CAP waveforms from LS and CON mice had similar shapes (Fig. 2B), with no significant differences in half-peak width and peak-to-peak amplitude (Fig. 2C).

### 3.2 Increased baseline activity of the vagus nerve in the LS group

**Fig. 3:**
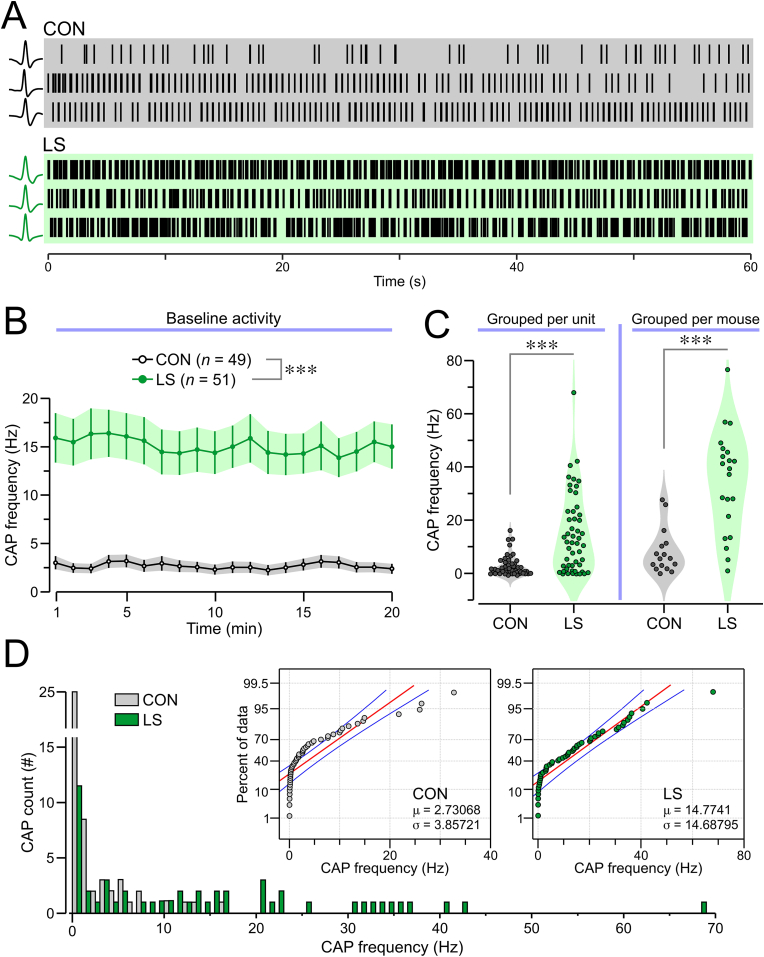
Baseline activity of the vagus nerve. **(A)** Raster plots showing 3 representative recordings per group; insets (*at left*) display the associated CAP waveforms. **(B)** Time series for CAP frequency (mean ± SEM, 1-min bins) showing that LS mice have significantly higher values than CON mice for the entire baseline period (∗∗∗*P* < 0.0001, 2-way ANOVA). **(C)** Violin plots of CAP frequency confirm that the LS group has much higher firing rate, calculated per unit (*left,* ∗∗∗*P* < 0.0001, KS test) and per mouse (*right,* ∗∗∗*P* < 0.0001, KS test). **(D)** Histogram showing the mean CAP frequency for each unit and cumulative probability plots for CON and LS groups (note the different scales for the X axes for the two groups); μ,mean, σ, standard deviation.

During the initial 20-min BL, units from LS mice showed significantly higher activity compared to CON mice (Fig. 3A–C). This increased activity was evident both when analyzing individual CAPs (Fig. 3C *left*, mean ± SEM, CON = 5.79 ± 0.9 CAPs/s, LS = 14.22 ± 1.85 CAPs/s; *P* < 0.001, KS test) and when summing all vagal activity for each mouse (Fig. 3C *right*, CON = 18.84 ± 3.51 CAPs/s, LS = 36.24 ± 4.1 CAPs/s; *P* = 0.015, KS test). The distribution of BL firing rates showed that while both groups had right-skewed distributions, LS mice had a much higher fraction of vagal units with elevated firing rates, with many LS units reaching the 20–70 Hz range (of note, zero CON units reached this level of high firing) (Fig. 3D).

**Fig. 4:**
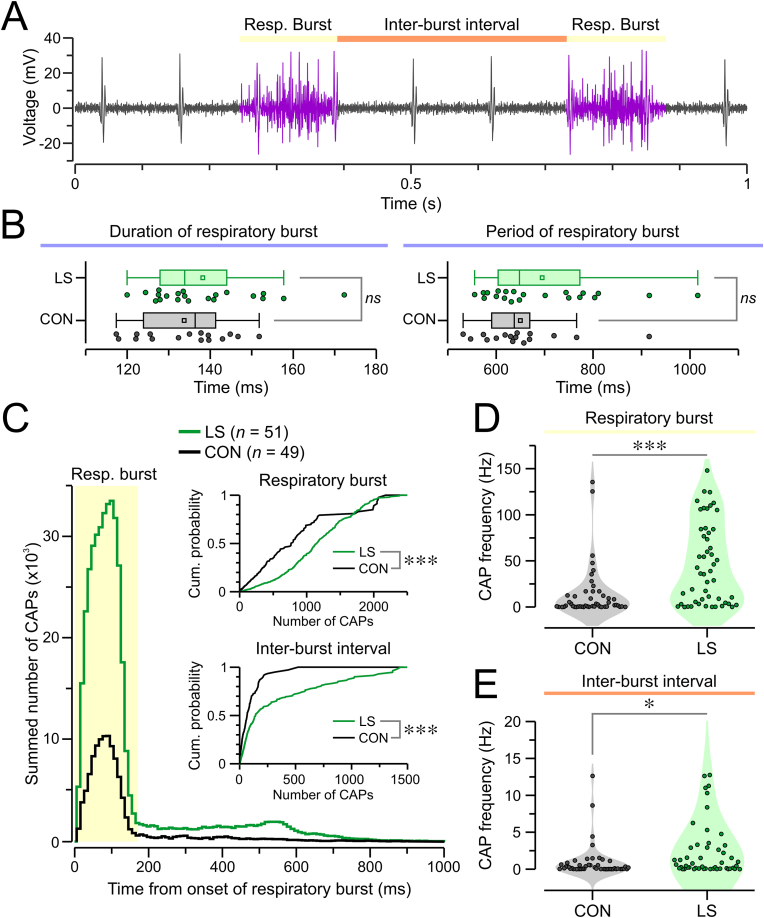
Respiratory modulation of the activity of the vagus nerve. **(A)** Representative recording (1-s epoch) showing respiratory bursts in purple and inter-burst intervals in black. **(B)** Box-and-whisker plots showing that LS and CON groups have respiratory bursts of similar duration (*left*, *P* = 0.271, *t*-test) and period (*right*, *P* = 0.342, MW test). Each plot displays the mean (square), median (line), inter-quartile range (box), and 10–90 range (whiskers), each dot represents a mouse. **(C)** Peri-event histogram showing the summed number of CAPs across CON or LS mice aligned from the start of each respiratory burst (“Resp. burst”). The highlighted portion depicts the time corresponding to the average duration of respiratory events. *Inset,* cumulative probability plots showing that LS mice have significantly higher number of CAPs during respiratory bursts (*top*, ∗∗∗*P* < 0.0001, KS test) and inter-burst intervals (*bottom*, ∗∗∗*P* < 0.0001, KS test). **(D)** Violin plot for the CAP frequency during respiratory bursts for CON and LS units, each dot represents one CAP unit (∗∗∗*P* < 0.0001, KS test). **(E)** Same as (D) but for CAP frequency during inter-burst intervals (∗*P* < 0.022, KS test). (For interpretation of the references to color in this figure legend, the reader is referred to the Web version of this article.)

Given the respiratory modulation of vagus nerve activity, we examined whether LS affected activity within respiratory bursts and during the inter-burst intervals (Fig. 4A). The vagus nerve has been shown to carry such robust respiratory signals that they can serve as reliable markers for respiration, as the respiratory-related vagal profiles closely mirror respiratory cycles measured by conventional respiratory measurements (Sevcencu et al., 2018). The duration and period of respiratory bursts were similar between groups (Fig. 4B, duration: CON = 133.73 ± 2.67 ms, LS = 138.2 ± 12.94 ms; *P* = 0.27; period: CON = 649.75 ± 92.8 ms, LS = 694.87 ± 26.6 ms; *P* = 0.23, *t*-test). To visualize the relationship between respiratory activity and CAPs, we created peri-event histograms aligned to the start of each respiratory burst. We found that LS units fired significantly more CAPs (during bursts and inter-burst intervals) when compared to CON units (Fig. 4C, burst: CON = 140.24 ± 12.3 CAPs, LS = 482.09 ± 21.47 CAPs, *P* < 0.001; inter-burst: CON = 4.74 ± 0.33 CAPs, LS = 18.96 ± 1.21 CAPs, *P* < 0.001, KS test). CAP frequency was also significantly higher in LS mice during both respiratory bursts (Fig. 4D, CON = 13.09 ± 3.96 CAPs/s, LS = 46.24 ± 6.17 CAPs/s; *P* < 0.001, KS test) and inter-burst intervals (Fig. 4E, CON = 0.88 ± 0.32 CAPs/s, LS = 3.52 ± 1.28 CAPs/s; *P* = 0.022, KS test).

**Fig. 5:**
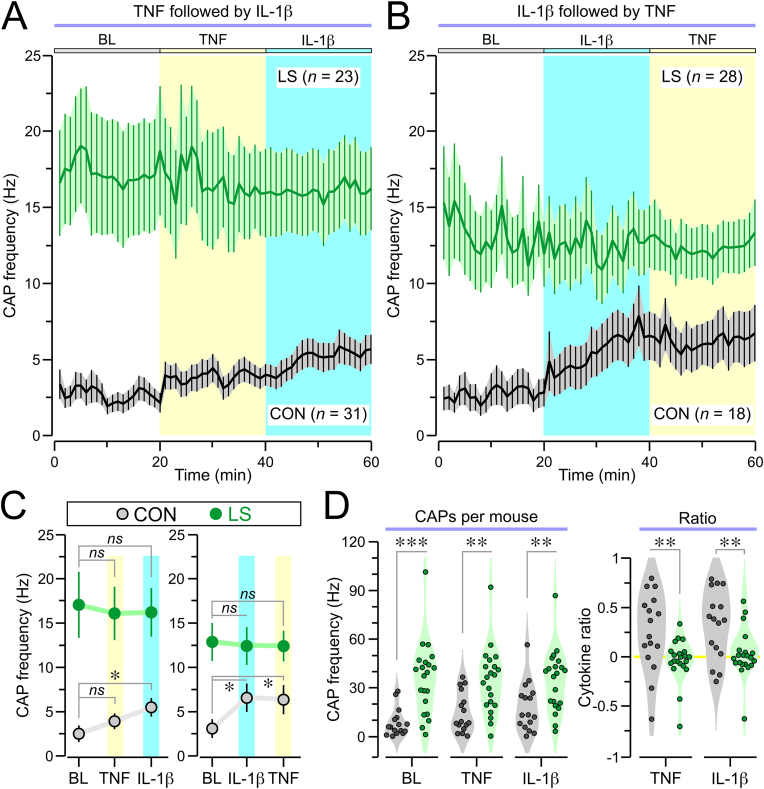
Vagal activity in response to cytokine injections. **(A)** Time series for CAP frequency (mean ± SEM, 1-min bins) of all units recorded in CON and LS mice. A baseline (BL) period is followed by injection of TNF (at 20 min) and injection of IL-1β (at 40 min). **(B)** Same as (A) but IL-1β is injected at 20 min and TNF at 40 min. **(C)** Plots showing the CAP frequency (mean ± SEM) for the last 10 min of BL, TNF, and IL-1β periods; *left,* TNF followed by IL-1β (CON: BL v. TNF, *P* = 0.06; BL v. IL-1β, ∗*P* = 0.02; LS: BL v. TNF, *P* = 0.929; BL v. IL-1β, *P* = 0.826; MW test). *Right,* IL-1β followed by TNF (CON: BL v. IL-1β, ∗*P* = 0.041; BL v. TNF, ∗*P* = 0.034; LS: BL v. IL-1β, *P* = 0.967; BL v. TNF, *P* = 0.941; MW test). **(D)***Left,* violin plots showing the summed frequency of all CAPs from each mouse for the last 10 min of BL (∗∗∗*P* < 0.0001, MW test), TNF (∗∗*P* = 0.001, MW test), and IL-1β (∗∗*P* = 0.008, *t*-test). *Right*, violin plots showing the cytokine ratios (defined in Materials and Methods) for TNF (∗∗*P* = 0.001, MW test), and IL-1β (∗∗*P* = 0.008, MW test). Each dot represents a mouse.

### 3.3 Dysregulated response to cytokines by the vagus nerve in the LS group

We examined whether the response of the vagus nerve to intraperitoneally injected cytokines was altered by LS. We established a stable recording (20 min BL), which was followed by administration of the first cytokine (at 20 min) and subsequent injection of the second cytokine (at 40 min). The experiments were performed in a counterbalanced manner with respect to the sequence of TNF and IL-1β, so that roughly half the mice received TNF at 20 min and IL-1β at 40 min (Fig. 5A), and the other half received IL-1β at 20 min and TNF at 40 min (Fig. 5B). The results showed that the elevated activity of LS units at BL remained high following cytokine challenges and, when examined as population averages across units, the post-cytokine periods appeared indistinguishable from pre-cytokine periods (Fig. 5A and B). This contrasted to the findings in CON mice in which there was a clear increase in activity following cytokine injections (Fig. 5A and B, Supplemental Fig. 1).

We compared the CAP frequency during the 20-min BL period for each vagal unit to the 20-min periods following injection of each cytokine. When TNF was administered first and IL-1β was given second, the CAP frequency for CON units showed a trend for increased activity following TNF (Fig. 5C *left, P* = 0.06, MW test) and a significant increase after IL-1β (Fig. 5C *left, P* = 0.002, MW test). In contrast, LS units showed no significant changes in the CAP frequency when comparing BL, TNF, and IL-1β periods (Fig. 5C *left*). When IL-1β was administered prior to TNF, the CAP frequency of CON units showed a significant increase during IL-1β compared to BL (Fig. 5C *right, P* = 0.041, MW test), and a continuous elevation during the later addition of TNF, which was also significantly higher than BL (Fig. 5C *right, P* = 0.034, MW test). LS units showed no modulation of CAP frequency following injection of either cytokine, although their values were higher than CON units for every condition (Fig. 5C *right*).

Moreover, we compared the total CAP frequency on the basis of individual animals for the BL, TNF, and IL-1β periods regardless of which cytokine was administered first, and found that for all 3 conditions, CAP frequency was significantly higher in LS mice when compared to CON mice (Fig. 5D). We used the total CAP frequencies to calculate the ratio of activity for the cytokine responses compared to BL (for each mouse). Strikingly, this revealed high ratios for both TNF and IL-1β in CON mice but significantly lower ratios for both cytokines in LS mice (Fig. 5D, TNF, *P* = 0.001; IL-1β, *P* = 0.008, MW test). Together, these findings show that at the level of population means, the response to cytokines is abrogated in LS mice, and that the vagal activity in LS mice is higher than CON mice regardless of cytokine challenge.

### 3.4 Firing dynamics of individual vagal units

**Fig. 6:**
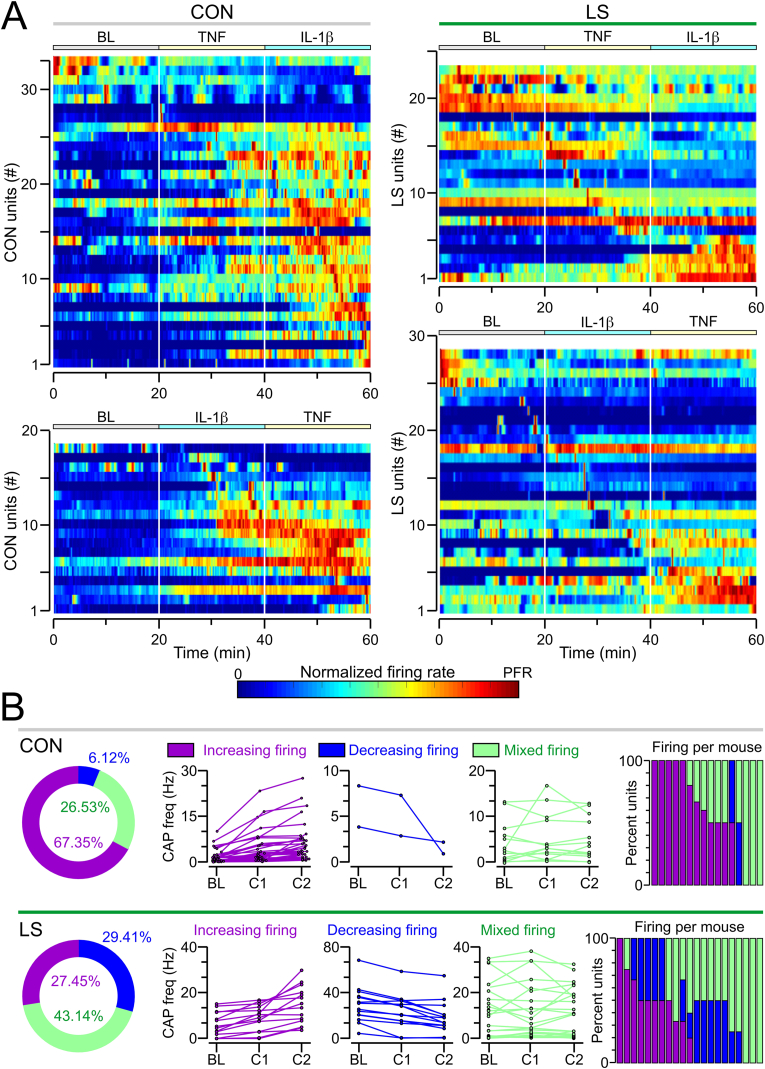
Firing rate dynamics for the vagal units. **(A)** Heatmaps of normalized firing rates for single vagal units in CON (*left*) and LS (*right*) mice. Each row of a heatmap represents a vagal unit and each column a 10-s bin. The units are ordered from top to bottom according to the time they exhibit their peak firing rate (PFR). Each heatmap is separated into baseline (BL), TNF and IL-1β periods (each lasting 20 min) by vertical white bars. **(B)** Each vagal unit is categorized into one of 3 categories: units that increase their firing after each cytokine injection, units that decrease their firing after each cytokine injection, or units which have a mixed response, defined as an increase to one cytokine and a decrease to the other cytokine regardless of the order in which each is injected. *Left,* donut plot showing the percent of units which fall into each category. *Middle,* line-series plots showing the frequency of firing for each vagal unit during the BL period, following injection of the first cytokine (C1), and the second cytokine (C2), regardless of which cytokine is actually administered. *Right,* stacked histograms showing the percent of vagal units which fall into each category for each mouse.

To test whether the responses to injected cytokines were masked in LS mice (due to their elevated BL firing rate), we examined the firing dynamics of each unit separately to check whether we could discern a cytokine response. We normalized the activity of each unit, so that it ranged from no activity (‘0’) to its peak firing rate (‘1’). Fig. 6A shows the vagal units, ordered from top to bottom by the time at which the peak firing rate occurred for each unit, and reveals that in CON mice, most vagal units showed the lowest relative activity during the BL period, and that discernible sub-populations of units increased their firing rates following administration of each cytokine (Fig. 6A *left*). In LS mice, it was evident that some sub-populations responded to cytokine injections with an increased firing rate, but notably, many more units responded with decreased firing rates (Fig. 6A *right*).

To quantify this phenomenon, we categorized all units based on their cytokine response, regardless of the order in which the cytokines were administered. Units that increased their firing in response to both cytokines were categorized as ‘*increasing-firing’*, units that decreased their firing in response to both cytokines as ‘*decreasing-firing’*, and units that increased their firing to one cytokine but decreased to the other as ‘*mixed-firing’*. Notably, the majority of units recorded from CON mice belonged to the increasing-firing category (67.35 %), such that their peak firing rates tended to occur at the tail of the 60-min experiment (Fig. 6B). The second most common category for CON units was mixed-firing (26.53 %), and a small percent were decreasing-firing (6.12 %). Remarkably, units recorded from LS mice had substantially more variable firing patterns (Fig. 6B), with mixed-firing being the most common category (43.14 %), followed by decreasing-firing (29.41 %) and increasing-firing (27.45 %). When analyzed on an animal-by-animal basis, it became apparent that at least half of the CON units were increasing-firing units, but several LS mice had no increasing-firing units at all (Fig. 6B *right*). These findings highlight the substantial alteration in response dynamics to cytokines by the vagus nerve in LS mice.

### 3.5 Altered signal discriminability in the vagus nerve of LS mice

**Fig. 7:**
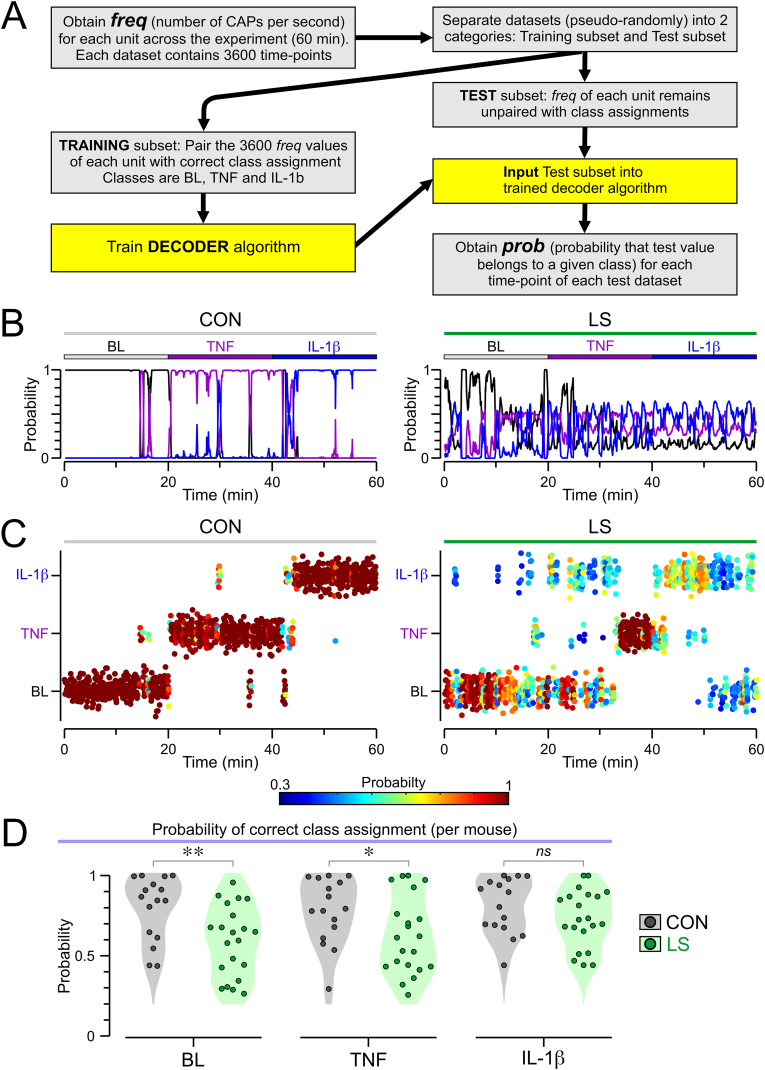
Bayesian decoding based on vagal activity. **(A)** Schematic of the decoding procedure. **(B)** Examples from CON and LS mice showing each decoded timepoint and the probability of prediction for each timepoint to be categorized as one of the 3 possible classes: baseline (BL, black), TNF (purple), or IL-1β (blue). **(C)** Examples of decoded recordings shown as dot plots in CON and LS mice. Each decoded timepoint is represented as a dot assigned to the class with the highest probability (among the 3 possible classes): BL (bottom of each plot), TNF (middle of each plot), or IL-1β (top of each plot). **(D)** Violin plots showing the probability of correct class assignment in each of the 3 classes for CON and LS mice: BL (*left,* ∗∗*P* = 0.008, MW test), TNF (*middle*, ∗*P* = 0.046, *t*-test), and IL-1β (*right, P* = 0.183, *t*-test) periods; each dot represents a mouse; *ns*, non-significant. (For interpretation of the references to color in this figure legend, the reader is referred to the Web version of this article.)

We questioned whether LS could lead to disorganization of the immune-encoding properties of the vagus nerve. Our data showed that when cytokines were administered in the periphery, the state of the signal carried through the vagus nerve was altered during LS. We reasoned that this quality could be exploited by using machine learning approaches to predict the immune status of the animal based on the activity of the vagus nerve, but this requires an intact neural code. Therefore, we used a naïve Bayes decoder to probe the integrity of the signal transmitted by the vagus nerve in CON and LS mice. Fig. 7A shows a flowchart for the decoding process that started by establishing a time series dataset of the CAP frequency at every second (for each recording), which was then pseudo-randomly split into two subsets (‘training’ and ‘test’). The training subset consisted of CAP frequencies paired with the correct classification (BL, TNF, IL-1β) for each timepoint. The test subset only featured CAP frequencies. The training subset was used to train the decoder algorithm, and the test subset was passed into the trained algorithm (Fig. 7A). For each timepoint of the test subset, the algorithm assigned probabilities for each of the 3 possible classes in the CON and LS groups (Fig. 7B). After that, the class with the highest probability was chosen as the decoded class in the CON and LS groups (Fig. 7C).

We compared the probability of assigning the correct class for each of the 3 classes. The decoder had a lower probability of predicting the correct class in LS mice for the BL period (Fig. 7D, *P* = 0.008, MW test) and after injection of TNF (Fig. 7D, *P* = 0.046, *t*-test). There was no significant difference for IL-1β (Fig. 7D, *P* = 0.183, *t*-test). The decoded recordings for individual mice are included, as dot plots, in the supplemental information (Supplemental Fig. 2 for CON mice, Supplemental Fig. 3 for LS mice). These findings demonstrate that vagal activity patterns in LS mice are less discriminable between baseline and cytokine-induced states, suggesting altered neural signaling in response to immune challenges.

## 4 Discussion

The objective of this study was to describe the vagus nerve's baseline state and immune surveillance during long sepsis in male mice. Unlike the highly inflammatory state observed in acute sepsis, which is characterized by high amounts of cytokine release including TNF and IL–6, LS represents a distinct chronic condition with persistent pathophysiological changes that extend well beyond the resolution of the initial septic event (Strohl et al., 2024; Valdés-Ferrer et al., 2013). Our investigation revealed profound alterations in vagus nerve function six weeks after CLP, a model for LS. Using cervical vagus nerve recordings with cuff electrodes (Steinberg et al., 2016), we acquired CAPs from LS and control mice, successfully isolating distinct neural units with advanced clustering algorithms (while eliminating cardiac signals). A striking result was that the baseline vagal firing was dramatically elevated in LS mice (14 CAPs/s per unit) when compared to the relatively quiescent baseline firing of control mice (6 CAPs/s per unit). This hyperactivity was consistent when measured at both individual unit and whole-animal levels. Of note, many LS vagal units reached firing frequencies of 20–70 Hz, which was a range never observed in control animals. Moreover, respiratory analysis indicated that LS mice showed markedly increased vagal firing during both respiratory bursts and inter-burst intervals, pointing to a global elevation of vagus nerve activity in LS throughout the respiratory cycle.

Importantly, LS mice displayed a blunted response to inflammatory cytokine challenges. While control mice had the expected pattern of increased vagal firing following systemic administration of TNF and IL-1β (Silverman et al., 2018; Steinberg et al., 2016; Zanos et al., 2018), LS mice showed no discernible change in their already elevated firing patterns in response to cytokines, suggesting a profound dysregulation of immune sensing. This study also introduced an innovative analysis by examining the firing dynamics of individual vagal units, which revealed a significant shift in response patterns. While control mice predominantly showed increased firing after cytokine exposure (67 % of units), LS mice displayed much more variable responses, with many units actually decreasing their firing rates after cytokine administration. This counterintuitive response suggested a malfunction of the inflammatory reflex. Indeed, only 28 % of LS vagal units showed the normal increasing-firing pattern, with some LS mice having no increasing-firing units whatsoever.

Using a naïve Bayes decoder to assess the integrity of the signal carried through the vagus nerve, this study found that the activity patterns in LS mice were significantly less distinguishable between conditions compared to controls, particularly during baseline (*P* = 0.008) and following TNF administration (*P* = 0.046). The elevated baseline activity in LS mice showed characteristics similar to cytokine-induced responses in healthy mice (Silverman et al., 2018; Steinberg et al., 2016), making it difficult to distinguish between baseline and post-cytokine states. While the decoder could identify IL-1β-related signals in LS mice, this likely reflected the distinct pattern of decreased activity following IL-1β administration (Fig. 6A), contrasting with the increased activity in control mice. These computational findings, while not direct measures of neural encoding, suggest substantial alterations in vagal activity patterns following sepsis. The reduced signal discriminability, combined with the observed hyperactivity and altered cytokine responses, indicates a fundamental disruption of normal vagal signaling patterns in LS. Together, these findings reveal that sepsis induces a state of chronic vagal hyperactivity paired with paradoxical insensitivity to acute immune challenges.

The role of the vagus nerve in sepsis has been examined using stimulation or vagotomy (Borovikova et al., 2000; Huston et al., 2007; Kessler et al., 2012; Kox et al., 2012; Mac et al., 2025; Rana et al., 2018), but neurophysiological recordings from the intact vagus nerve represent an exciting current approach. Early recordings of vagal afferents suggested that IL-1β activated vagal fibers (Ek et al., 1998; Niijima, 1992, 1996; Niijima et al., 1995; Watkins et al., 1995). The study by Steinberg et al. (2016) recorded vagal CAPs and established unequivocally that the increased activity after administration of TNF and IL-1β corresponded to activation of afferent fibers. Zanos et al. (2018) showed that the vagus nerve carried discriminable cytokine-specific signals, and that a Bayes decoder predicted which cytokine was injected based on the recorded CAPs. Several reports have introduced advanced recording techniques, such as high-density carbon-fiber microelectrode arrays (Jiman et al., 2020), flexible thin-film microchannel arrays (Lim et al., 2024), carbon nanotube yarn biosensors (Marmerstein et al., 2022), and wireless recording and stimulation systems (Wright et al., 2022). Recently, Okonogi et al. (2024) performed vagus nerve recordings and stimulations in freely moving mice and showed that the spike rates of the cervical vagus nerve changed dramatically depending on the anxiety state in stress-resilient mice (measured in the elevated plus maze task), but vagal activity did not change in stress-susceptible mice. Remarkably, the measured spike rates in behaving mice ranged from 1 to 15 Hz, which are similar values to those obtained in this study from anesthetized mice.

A fascinating development has been the advent of neurophysiological recordings from the cervical vagus nerve in awake human subjects (Farmer et al., 2025; Ottaviani et al., 2020; Patros et al., 2022, 2024, 2025). The initial study by Ottaviani et al. (2020) used ultrasound-guided insertion of tungsten microelectrodes into fascicles of the vagus nerve and recorded tonically active units that fired in relation to cardiac intervals as well as presumed sensory axons from the airways and motor axons to the larynx. Interestingly, Patros et al. (2022) found that human vagal activity displayed a strong respiratory modulation, such that large bursts of multi-unit activity occurred during periods of inspiration, especially during slow deep breathing. This human result aligns well with our finding of intense vagal activity during respiratory events in anesthetized mice (Fig. 4).

A fundamental issue raised by this study is the disruption of inflammatory reflex homeostasis and vagal sensing of cytokines in long sepsis. Our findings suggest that the LS condition substantially alters vagus nerve firing patterns, shifting from a responsive surveillance mode to a chronically hyperactive state that disrupts the immune coding properties of the sensory vagus nerve. The blunted cytokine responses observed in LS mice align with the long-term immunosuppression experienced by sepsis survivors, indicating a risk factor for subsequent infections (Shankar-Hari and Rubenfeld, 2016). Although HMGB1 may remain somewhat elevated at this timepoint, it is unlikely that this alone could account for the dramatic increase in vagal firing we observed, particularly given that the vagus nerve remains capable of discriminating between different cytokines when they are administered acutely (Zanos et al., 2018). Instead, our findings suggest a fundamental maladaptation in vagal signaling, possibly involving altered changes in neural processing at the level of the nucleus tractus solitarius.

While the exact molecular mechanisms remain unclear, we propose that, following sepsis, the vagus nerve remains in an equilibrium characteristic of the disease state. This concept aligns with our decoder analysis, which revealed significantly impaired discrimination of inflammatory signals in LS mice. This interpretation is supported by recent work demonstrating similar alterations in vagal firing patterns in individual nodose sensory neurons during peak colitis (Huerta et al., 2025). The parallel findings of increased baseline activity with altered responses to inflammatory markers suggest a conserved neurophysiological response to persistent inflammation across different disease models. The dysregulated neural activity represents a pathologically altered regulatory mechanism that fails to properly modulate immune responses, as described in the inflammatory reflex model (Tracey, 2002; Pavlov and Tracey, 2012). This maladaptive recalibration may perpetuate chronic sepsis sequelae, suggesting that future studies could explore neuromodulation of the vagus nerve as a strategy to restore vagal homeostatic signaling. Indeed, vagus nerve stimulation is currently being explored as a treatment paradigm for chronic inflammatory diseases such as inflammatory bowel disease and Crohn's disease (Bonaz et al., 2017; Sinniger et al., 2020). While our study focused on responses to cytokines, future studies using non-cytokine stimuli would be valuable in determining whether these alterations are specific to inflammatory signals or represent a more general change in vagal signaling properties.

A limitation to this study is the lack of an awake preparation for vagus nerve recordings. While this report and others (Masi et al., 2019; Silverman et al., 2018; Steinberg et al., 2016; Zanos et al., 2018) have successfully recorded the vagus nerve in anesthetized mice, upcoming studies should take advantage of newly developed techniques to obtain chronic recordings in freely moving animals (Okonogi et al., 2024; Wright et al., 2022). Another limitation is our focus on a single chronic time point (6 weeks post-septic shock), which prevents determination of whether the observed vagal dysfunction emerges during acute sepsis or develops gradually during recovery. Future studies examining multiple time points would be valuable to establish the temporal trajectory of these neurophysiological alterations. An additional limitation of this study is the use of cuff electrodes (encircling the entire nerve) that can easily detect CAPs but not individual axons potentials, which are often below the noise floor of standard cuff electrode recordings. The use of specialized probes that directly access the nerve fascicles, such as interfascicular electrodes (longitudinal or transverse), together with enhanced signal processing (spike sorting algorithms and machine learning classification) could achieve reliable single axon recordings of the vagus nerve. Finally, our study focused exclusively on cytokine challenges; investigation of non-cytokine stimuli would help determine whether these alterations are specific to inflammatory signals or represent broader changes in vagal signaling properties.

## 5 Conclusion

In conclusion, six weeks after a septic shock, the vagus nerve shows higher baseline activity and diminished response to peripheral cytokines. Most strikingly, the ability to accurately decode vagus nerve signals is significantly reduced during baseline and during periods of cytokine administration. Our findings suggest a profound dysregulation of neural signaling and the neuroimmune axis during long sepsis. Future studies may explore whether targeting vagus nerve function could help address the chronic sequelae of sepsis.

## CRediT authorship contribution statement

**Joshua J. Strohl:** Conceptualization, Data curation, Formal analysis, Investigation, Methodology, Project administration, Resources, Software, Validation, Visualization, Writing – original draft, Writing – review & editing. **Tomás S. Huerta:** Conceptualization, Formal analysis, Investigation, Methodology, Writing – review & editing. **Sergio Robbiati:** Conceptualization, Investigation, Methodology. **Patricio T. Huerta:** Conceptualization, Data curation, Formal analysis, Funding acquisition, Investigation, Methodology, Project administration, Resources, Software, Supervision, Validation, Visualization, Writing – original draft, Writing – review & editing.

## Data availability

The authors declare that all the datasets supporting the findings of this study are displayed within the manuscript and supplemental figures. Datasets will be made available upon reasonable request. The spike sorting algorithm used can be found at: http://public.feinsteininstitute.org/cbem/PNAS%20Submission/.

## Funding

Funding was provided by the 10.13039/100000002National Institute of Health (10.13039/100000002NIH) grant 5P01AI102852 (to PH) and 10.13039/100000002NIH grant 5P01AI073693 (to PH), as well as the 10.13039/100000005Department of Defense (10.13039/100000005DOD) impact award W81XWH1910759 (to PH) and impact award HT9425-24-1-0976 (to PH).

## Declaration of competing interest

The authors declare no competing interests.

## Data Availability

The data that has been used is confidential.
