# Supplementary material for: Dysregulated neural coding in the vagus nerve during long sepsis
Source: Brain Behav Immun Health. 2025 Jun 19;47:101043. doi: 10.1016/j.bbih.2025.101043 (PMC12226043; doi:10.1016/j.bbih.2025.101043)
Supplement: Multimedia component 1 [file mmc1.pdf]

Dysregulated neural coding in the vagus nerve during long sepsis

Joshua J. Strohl, Tomás S. Huerta, Sergio Robbiati, Patricio T. Huerta

SUPPLEMENTARY MATERIAL

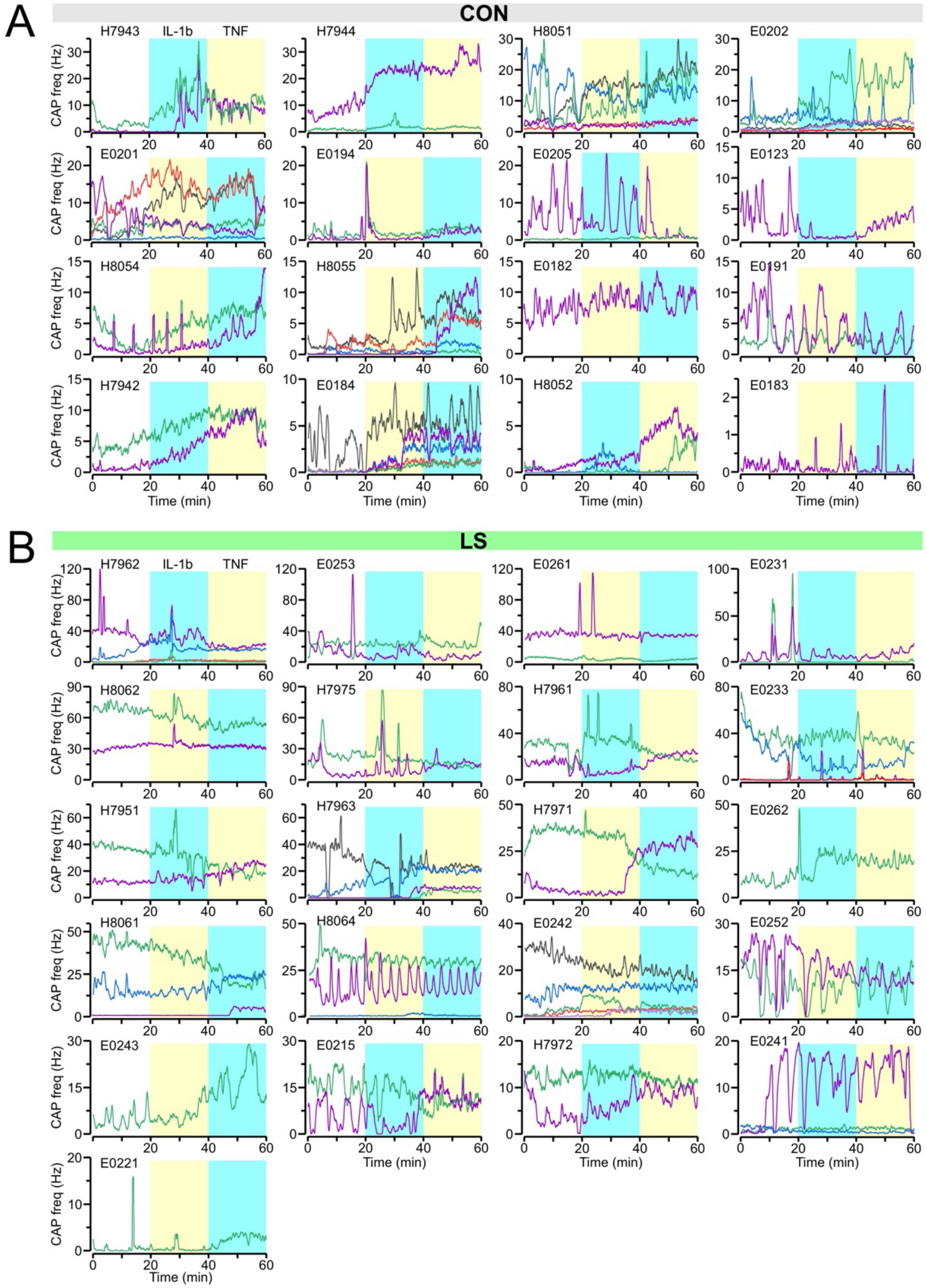

Supplemental Fig. 1: CAP frequency traces for each mouse. **(A)** CAP frequency for every unit recorded in CON mice. Each plot represents a single mouse and each line represents the CAP frequency of a single unit across time. Baseline periods of the recordings are depicted with a white background, IL-1β periods with a blue background, and TNF periods with a yellow background. **(B)** Same as for (A), but for LS mice.

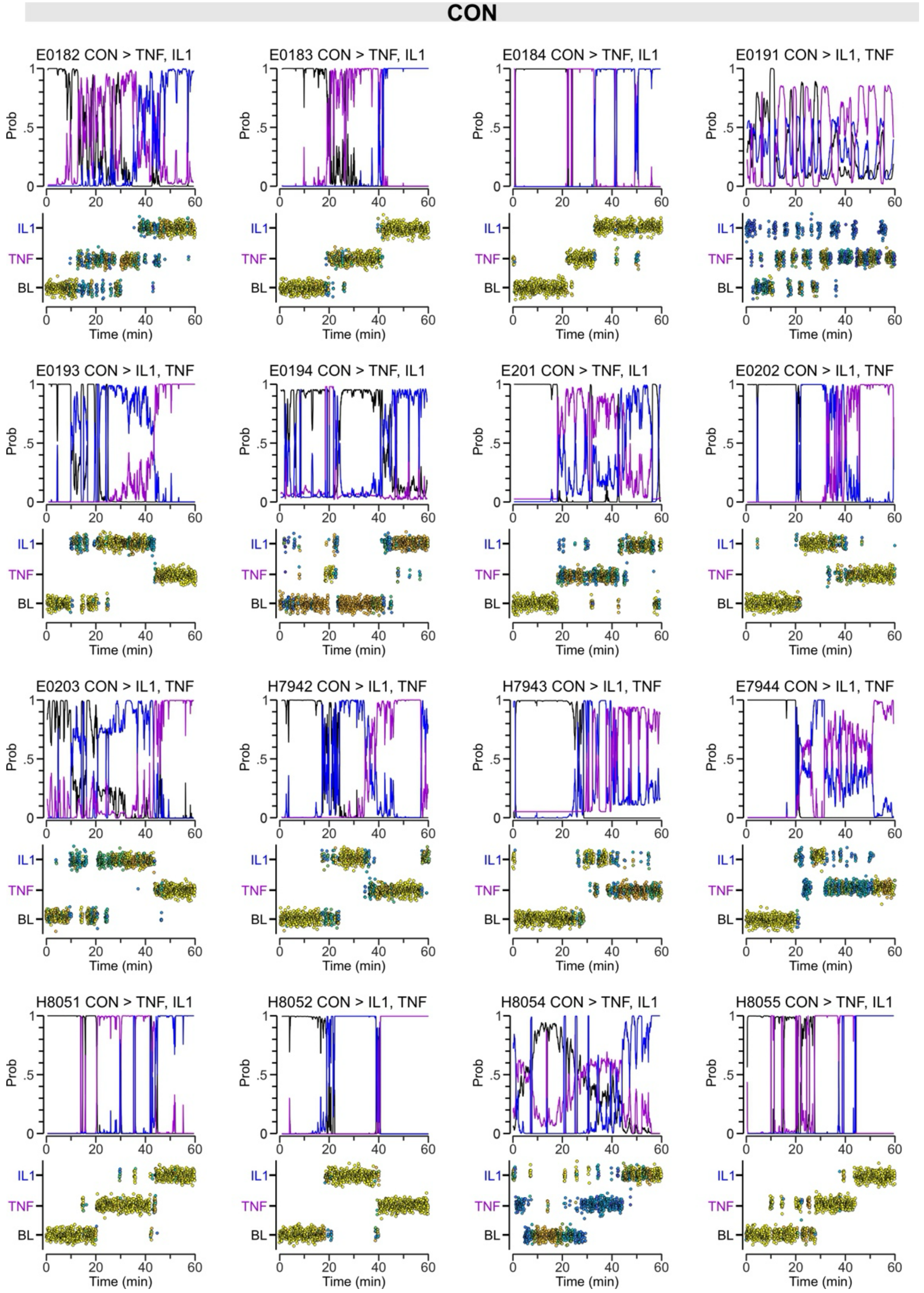

Supplemental Fig. 2: Bayesian decoding probabilities and class assignments for each control (CON) mouse. Line plots of each decoded timepoint and the probability of prediction to be categorized as 1 of the 3 possible classes: BL (black), TNF (purple), or IL-1β (blue) for all mice (top plot for each mouse). Plots of each decoded timepoint represented as a dot assigned to the class with the highest probability among the 3 possible classes: BL (bottom of each plot), TNF (middle of each plot), or IL-1β (top of each plot) for all CON mice.

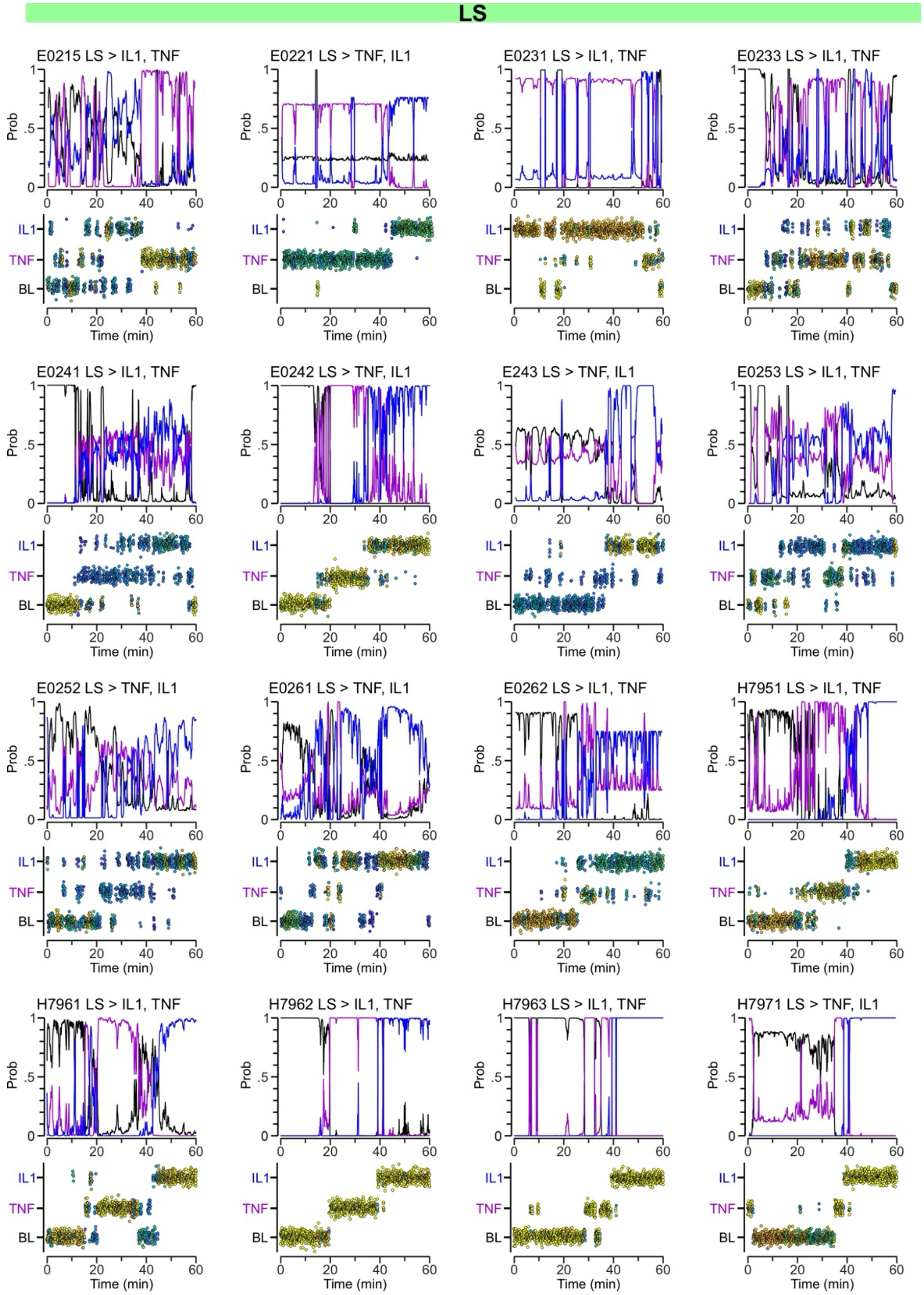

Supplemental Fig. 3: Bayesian decoding probabilities and class assignments for each long sepsis (LS) mouse. Line plots of each decoded timepoint and the probability of prediction to be categorized as 1 of the 3 possible classes: BL (black), TNF (purple), or IL-1β (blue) for mice (top plot for each mouse). Plots of each decoded timepoint represented as a dot assigned to the class with the highest probability among the 3 possible classes: BL (bottom of each plot), TNF (middle of each plot), or IL-1β (top of each plot) for LS mice.
